# Recent Trends in Synchronous Brain Metastasis Incidence and Mortality in the United States: Ten-Year Multicenter Experience

**DOI:** 10.3390/curroncol29110660

**Published:** 2022-11-02

**Authors:** Wenqiang Che, Jie Liu, Tengyue Fu, Xiangyu Wang, Jun Lyu

**Affiliations:** 1 Department of Neurosurgery, The First Affiliated Hospital of Jinan University, Guangzhou 510630, China; 2 Department of Clinical Research, The First Affiliated Hospital of Jinan University, Guangzhou 510630, China

**Keywords:** brain metastases, trends, incidence, mortality, SEER

## Abstract

Background: Large epidemiological studies describing the trends in incidence rates and mortality of synchronous brain metastases (SBMs) are lacking. The study aimed to provide a comprehensive understanding of the changes in the incidence and mortality of SBMs over the previous ten years. Methods: Trends in the incidence of solid malignancies outside of the CNS in patients with SBMs and incidence-based mortality rates were assessed using data from the Surveillance, Epidemiology, and End Results database. Joinpoint analyses were used to calculate annual percent changes (APCs) and 95% CIs. Results: Between 2010 and 2019, 66,655 patients, including 34,821 (52.24%) men and 31,834 (47.76%) women, were found to have SBMs, and 57,692 deaths occurred over this period. Lung cancer SBMs, melanoma SBMs, and breast cancer SBMs were ranked in the top three, having the highest age-standardized incidence rates. The incidence of SBMs decreased significantly with an APC of −0.6% from 2010 to 2019, while the APC was 1.2% for lung cancer SBMs, 2.5% for melanoma SBMs, and 0.6% for breast cancer SBMs. The SBM mortality first experienced a rapid increase (APC = 28.6%) from 2010 to 2012 and then showed a significant decline at an APC of −1.8% from 2012 to 2019. Lung cancer SBMs showed similar trends, while melanoma SBM and breast cancer SBM mortality increased continuously. Conclusions: SBMs incidence (2010–2019) and incidence-based mortality (2012–2019) declined significantly. These findings can advance our understanding of the prevalence of SBMs.

## 1. Introduction

Based on various approaches, the estimates of the incidence proportion of brain metastases (BMs) range from 20% to 40% [1,2,3]. Numerous BMs present with neurological symptoms, which frequently result in significant cognitive, quality-of-life, and performance-status impairment [1,4]. Surgery, stereotactic radiosurgery (SRS), whole-brain irradiation (WBRT), hippocampal-sparing WBRT (hsWBRT), targeted treatments, and chemotherapy are some of the therapeutic options available for BMs [5,6,7]. BMs have a profound influence on the clinical course of individuals with systemic cancer. The prognosis of BMs is still poor, despite optimized treatment regimens, with synchronous BMs having a median survival of 2.9 months and metachronous BMs having a median survival of 3.4 months among elderly patients [8]. It is clear that the cost of diagnosing and treating BMs has grown significantly, affecting many different medical subspecialties and the healthcare system. Epidemiologic studies on the incidence and mortality of BMs are in great need.

The incidence of BMs is thought to be rising, not just as a result of improvements in the care of patients with primary malignancies and a decline in mortality, but due to technical developments in neuro-imaging [9,10]. It was observed that the annual age-adjusted incidence rate of BM hospitalization increased significantly between 1987 and 2006 [2]. However, no such data collected after 2006 could further support these conjectures. Until recently, research by Cagney et al. was thought to provide new evidence for the present epidemic situation of synchronous BMs in the United States (U.S.). The study indicated that during diagnosis, synchronous BMs were present in 2.0% of all cancer patients and 12.1% of those with metastatic disease [11]. The exploration of trustworthy prognosticators for the outcome prediction of synchronous BMs has also received significant research attention [12,13]. For better guiding customized therapy strategies and understanding cancer natural history, descriptive studies are crucial. Large epidemiological studies focusing on current trends in synchronous BM incidence and mortality are lacking because of a scarcity of data.

To overcome these limitations, the current study aimed to use SEER data from 2010 to 2019 to gain a comprehensive understanding of the incidence and mortality trends of synchronous BMs based on demographic and primary tumor features at the time of diagnosis.

## 2. Materials and Methods

### 2.1. Ethics Approval

The NCI SEER study is retrospective in nature, and the ethics committee waived consent due to the study’s anonymized data and guarantee of patient privacy.

### 2.2. Data Sources

For patients diagnosed with systemic malignant malignancy between 2010 and 2013, information on synchronous brain metastases at the time of primary tumor diagnosis was first available in 2016 [11,12,13]. The ten-year (2010–2019) accessible information on synchronous brain metastases (SBMs) better reflected the change in the epidemic pattern over the research period when the analyses were updated to incorporate more recent data released in April 2022 based on the November 2021 submission. The SEER dataset, with 9 population-based registries that include Connecticut, Iowa, New Mexico, Utah, Hawaii, Detroit, San Francisco–Oakland, Atlanta, and Seattle–Puget Sound, is the most commonly used file to analyze trends in incidence and mortality of malignancies in the U.S. [14]. Given that the data for 2019 have not been updated in the SEER-9 database (Table S1), we selected another SEER file with 17 registries (SEER-17) that covers around 26% of the U.S. population to conduct epidemiological analyses [15]. The database contains patient demographics; malignancy diagnosis; and brain, lung, liver, and bone metastasis information. SBM death information, provided by SEER registries, recorded in death certificates, was validated by National Center for Health Statistics.

Within the incidence-based mortality SEER-17 file, death certificate information records the features of malignancies at the time of diagnosis that could be applied to derive age-adjusted mortality rates based on incidence according to variable features (for example, histological type, T-Stage, and N-Stage). Similar to incidence, only ten-year (2010–2019) data are available to calculate incidence-based mortality rates. Furthermore, this time period constraint corresponds well with the cohort periods for incidence analyses in our investigation.

Based on the background mortality risk in the general population, relative survival analyses adjust the observed survival in the specific cohort to convert the cumulative observed survival into a relative survival [16]. Relative survival data were extracted using the SEER-17 incidence database. One- to nine-year relative survival rates for SBMs and lung cancer with SBMs, breast cancer with SBMs, and melanoma with SBMs diagnosed between 2010 and 2018 were listed.

### 2.3. Demographic Characteristics

The demographic characteristics included in this epidemiological analysis were age at diagnosis/death, sex, race, median household income (MHHI), and area distribution. The SEER-17 file divides adult patients (age ≥ 20) into different age groups using 5-year age intervals, which results in 13 age intervals (20–24, 25–30, … ≥ 80). To simplify the analysis, four age groups (20–39, 40–59, 60–79, and ≥ 80) were determined finally. Race was classified as white, black, and other (which included American Indian/AK Native and Asian/Pacific Islander). MHHI higher than 75,000/year was defined as high income. The residential area was separated between rural and urban areas, which included urban and suburban areas. Originally, these data were extracted from medical records and submitted to cancer registries, except for age at death, which was abstracted from death certificates. Participant exclusion criteria were as follows: (i) unidentified age; (ii) unidentified MHHI and residence area.

### 2.4. Tumor Characteristics

In principle, histologic types of SBMs are identified according to the primary tumor site, except for melanoma, which is identified via histology rather than the primary site [11]. The identification of histology and the tumor site was conducted with reference to International Classification of Diseases for Oncology, Third Edition (ICD-O-3). The detailed topography and morphology code are shown in Table S2 in the Supplement data. The TNM staging system 8th (2018–2019) and 7th (2010–2017) editions were used to determine the T-Stage and N-Stage classification. The tumor exclusion criteria were as follows: (i) non-invasive/in situ neoplasms; (ii) nonsolid tumors; (iii) tumors of the central nervous system; (iv) unknown primary tumor site; (v) diagnosed at autopsy or death certificate.

### 2.5. Data Analysis

SEER*Stat software (version 8.4.0) was used to generate age-adjusted incidence rates and incidence-based mortality rates based on the 2000 U.S. standard population and expressed per 100,000 person-years. Rate disparities were evaluated to assess how SBM rate reductions or increases may have influenced trends. In addition, the Tiwari 2006 modification was used to generate rate ratios with 95% confidence intervals (CIs). Joinpoint Regression Program (version 4.7) employs log-linear models to assess and fit incidence and incidence-based mortality changes for the best-fitting model, with annual percentage changes (APCs) being produced [17]. Long-term trends were estimated using SEER-17 registry data from 2010 to 2019, allowing us to obtain the fewest “joinpoints” to fit the data. The statistical significance of APCs and differences between APCs for two time periods were tested using the SEER*Stat program, as described by Kim HJ et al. [17]. To assess statistical significance, we applied multiple statistical tests and utilized a type I error rate of 5%.

## 3. Result

**Figure 1:**
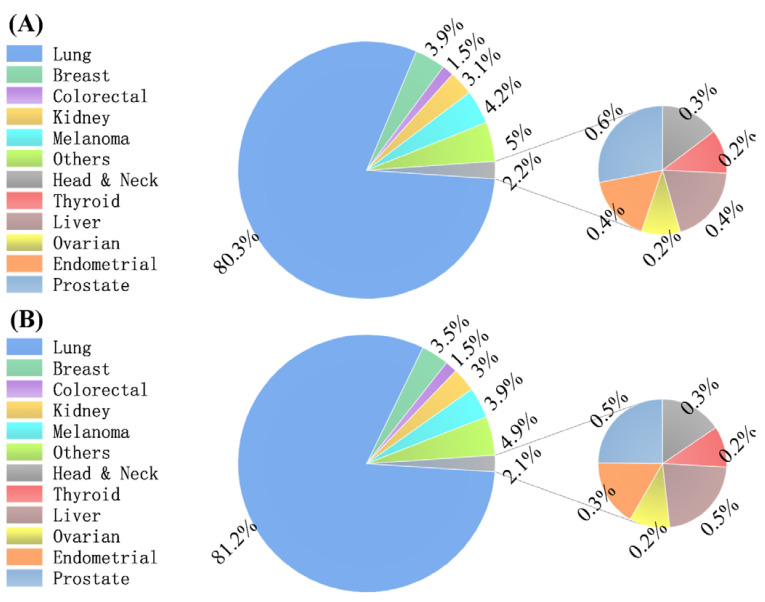
Distribution of primary cancer in patients with synchronous brain metastases in incidence analyses (**A**) and mortality analyses (**B**).

Of the 68,347 adult patients diagnosed with SBMs in the SEER-17 registries during 2010–2019, 66,655 (97.52%) fulfilled the inclusion and exclusion criteria and were eligible for entry into the study. Most of the participants were male (34,821, 52.24%) and white (53,257, 79.9%). The highest number of cases, 39,757 (59.65%), occurred in the group aged 60–79 years. A majority lived in metropolitan counties (84.91%), and 71.09% had an MHHI of less than 75,000/year. The most frequent primary tumor site was the lung (53,492, 80.25%), followed by melanoma (2812, 4.22%), breast (2571, 3.86%), kidney (2054, 3.08%), and colorectum (977, 1.47%) (Table 1, Figure 1A). Over half of SBM patients had received chemotherapy (33,383, 50.08%) and radiotherapy (44,520, 66.79%), and 5.53% had received surgical treatment. During the studied time period, the incidence-based mortality analysis showed 57,692 deaths of all causes. Of all the deceased patients, 53.14% were males, and 80.58% were white. Similarly to all cases, decedents tended to be older, be diagnosed with lung cancer SBMs (Table 1, Figure 1B), be more likely to live in an urban area, and have lower MHHI. Slightly more than half of the deaths had received no chemotherapy (30,458, 52.79%).

**Figure 2:**
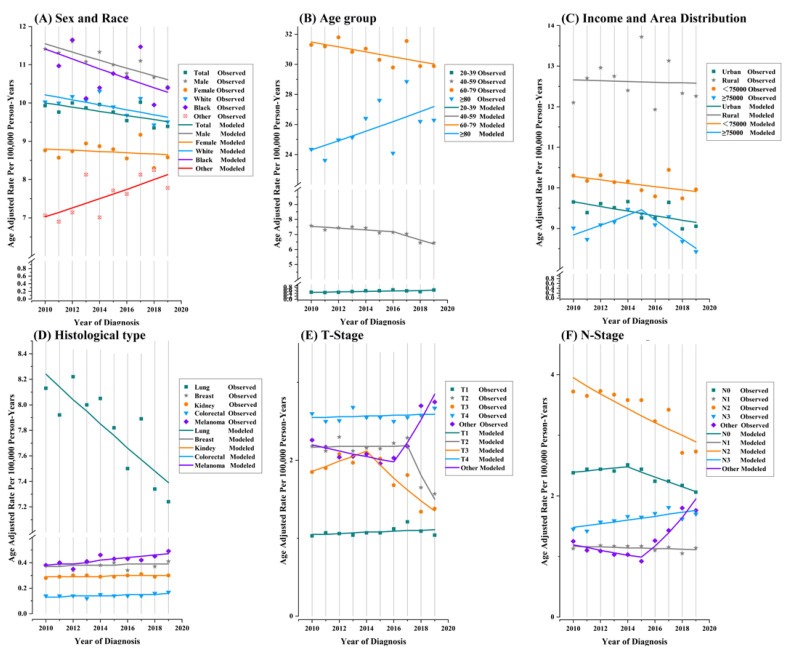
Trends in annual synchronous brain metastasis incidence rates by sex and race (**A**), age (**B**), median household income and area distribution (**C**), primary tumor site (**D**), T-Stage (**E**) and N-Stage (**F**). All rates presented were age-adjusted based on the 2000 U.S. standard population (cases per 100,000 person-years). Each segment on the line represents the annual percent change (APC).

We present trends in SBMs incidence by demographic and primary tumor characteristics at diagnosis in Table 2 and Figure 2. The change in the slope estimated using Joinpoint regression allowed us to obtain up to one join point for our study owing to the short time period studied. SBM incidence rates decreased significantly over the last decade at an APC of −0.6% (95% CI, −1.1 to 0; *p* < 0.001). SBM incidence rates showed a statistically significant decreasing trend for males, white people, those living in urban areas, those aged 40–59 years and 60–79 years, and patients receiving radiotherapy and surgical treatment, while non-statistically significant decreases were observed for females, black people, high-income families, rural-area patients, and patients without chemotherapy and surgery. SBM incidence rates increased among patients aged 20–39 years at a statistically significant APC of 2.8% and increased among patients older than 80 years at a non-statistically significant APC of 1.3%. SBM incidence for high-MHHI families increased by 1.4% (95% CI: −0.6 to 3.3) per year from 2010 to 2015, but it decreased by 2.6% during 2015–2019 (95% CI: −5.2 to 0.1). SBM incidence in males was 1.29 times higher than that in females, and the difference increased with age (from 0.96 (95% CI: 0.87 to 1.06) times in those aged 20–39 years to 1.38 (95% CI: 1.33 to 1.44) times in those older than 80 years) (Table S3). Overall, primary sites located in the lung, and head and neck revealed decreased SBM incidence during 2010–2019 with APCs of −1.2 (95% CI: −1.8 to −0.6) and −1 (95% CI: −4.1 to 2.1), and primary sites in breast, kidney, and melanoma demonstrated stable increases in incidence over time at APCs of 0.6 (95% CI: −1.3 to 2.5), 0.5 (95% CI: −0.2 to 1.2), and 2.5 (95% CI: 0.9 to 4.2), respectively.

**Figure 3:**
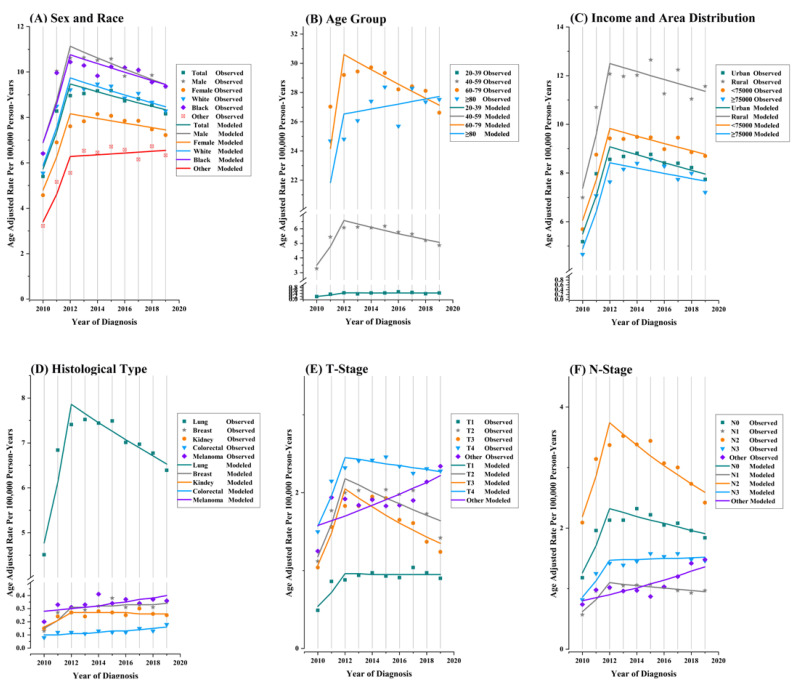
Trends in annual synchronous-brain-metastasis-incidence-based mortality rates by sex and race (**A**), age (**B**), median household income and area distribution (**C**), primary tumor site (**D**), T-Stage (**E**) and N-Stage (**F**). All rates presented were age-adjusted based on the 2000 U.S. standard population (cases per 100,000 person-years). Each segment on the line represents the annual percent change (APC).

The joinpoint analysis revealed that SBM-incidence-based mortality rates increased sharply at a positive APC of 28.6% (95% CI, 19 to 38.9; *p* < 0.001) between 2010 and 2012 (Figure 3, Table 3). Contrastingly, they steadily declined with a negative APC of −1.8 (95% CI, −2.8 to −0.8; *p* = 0.006) from 2012 to 2019. Similarly, a trend of first rising and then falling with a single joinpoint in 2012 was observed for both genders; white and black people, people aged 20–39, 40–59, and 60–79 years; both urban and rural-area patients; both low- and high-MHHI families; T1-, 2-, 3-, and T4-Stages; N0-, 1-, and N2-Stages; and patients with/without chemotherapy, with radiotherapy, and with/without surgical treatment. For SBM patients older than 80 years old, there was a rapid rise in incidence-based mortality rates (APC = 21.5; 95% CI, 5.4 to 40.2; *p* = 0.017), which then rose smoothly over time at an APC of 0.6% (95% CI, −1.3 to 2.6; *p* = 0.432). Trends in incidence-based mortality rates among patients receiving no radiotherapy also showed a rapid rise, followed by a gradual increase (Table 3, Figure S1). Men died at an incidence-based mortality rate around 1.18 times higher (95% CI: 1.16 to 1.19) than women, and the value reached 1.39 (95% CI: 1.34 to 1.45) among those over 80 years old (Table S3). By primary tumor site of SBMs, SBMs from melanoma (APC = 4.1; 95% CI, 0 to 8.4; *p* = 0.048) and colorectum (APC = 5.7; 95% CI, 2.3 to 9.3; *p* = 0.005) exhibited significantly increased mortality rates, while the mortality rates of breast cancer with SBMs demonstrated a rapid increase (APC = 44.8; 95% CI, 3.3 to 103; *p* = 0.037) from 2010 to 2012 and a stable increase (APC = 1.4; 95% CI, −3 to 6.1; *p* = 0.454) from 2012 to 2019. Additionally, the uptrend and downtrend of mortality rates of SBMs from lung (APC = 28.4 and 95% CI, 19.2 to 38.2; APC = −2.6 and 95% CI, −3.6 to−1.6) and kidney (APC = 30.1 and 95% CI, −4.1 to 76.7; APC = −0.6 and 95% CI, −4.6 to 3.6) were found in 2010 to 2012 and 2012 to 2019, respectively.

The analysis demonstrated that the relative survival rates for SBMs from lung, breast, and melanoma increased with the year of diagnosis (Table S4). The 1-year relative survival rate was 23.38% for SBM patients in 2010 and 34.45% for cases diagnosed in 2019, while the 5-year relative survival rate increased from 3.4% in 2010 to 5.24% in 2019. Despite some fluctuations, the 1-year relative survival rates for lung cancer with SBMs, breast cancer with SBMs, and melanoma with SBMs were variably ameliorated. During 2010–2014, the 5-year relative survival rate for melanoma with SMBs improved greatly (from 5.86% to 14.03%); lung cancer with SBMs improved slightly (from 2.95% to 4.56%), and breast cancer with SBMs fell from 13.4% to a low of 9.89%.

Tables S5–S12 in the Supplementary Material list the annual number of cases, fatalities, incidence rates, and incidence-based mortality rates. To investigate the differences between populations with SBMs and synchronous extracranial metastases (SEMs), we present the trends in incidence and incidence-based mortality rates of synchronous bone, liver, and lung metastases without SBMs in Tables S13–S15 and Figure S2 in the Supplementary.

## 4. Discussion

This is an advanced study to characterize trends in SBM incidence and mortality rates in the United States based on demographic and tumor characteristics at diagnosis.. Using the recently released 2010 to 2019 SEER registry file, we mainly found a significant downward trend in the age-adjusted incidence of SBMs from 2010 to 2019 (APC = −0.6; 95% CI, −1.1 to 0), and a first rising (2010–2012: APC = 28.6%; 95% CI, 19 to 38.9) and then a declining trend (2012–2019: APC = −1.8; 95% CI, −2.8 to −0.8) in incidence-based mortality, which was generally increasing from 2010 to 2019 (APC = 4.3; 95% CI, 2.8 to 5.8). Those findings showed similar trends in the changes in lung cancer SBM incidence and incidence-based mortality, while trends in breast cancer SBMs, melanoma SBMs, kidney cancer SBMs, and colorectal cancer SBMs had varied characteristics.

The SEER program suggests arenas for future epidemiological studies of SBMs, which were hardly going forward to determine the exact incidence before 2016, since reporting BMs was not mandated by local and federal registries [18]. As mentioned in the literature review, 2.03% of all solid cancer patients and 2.08% of midlife patients had synchronous BMs [11,12]. Lung cancer SBMs, breast cancer SBMs, renal cancer SBMs, melanoma SBMs, and colorectal cancer SBMs ranked as the top five in terms of the number of patients in the entire cohort [11]. Using the SEER database, several studies evaluated risk factors for mortality in patients with SBMs as well as risk factors for the development of SBMs [8,11,12,13,19]. Furthermore, a wide variety of novel prognostic nomograms for predicting the survival of SBMs from other systems were developed and validated based on the SEER database [20,21]. Unlike those epidemiologic studies, which reported the incidence proportion of BMs among cancer patients or subsets with metastatic disease during a specific time period, Singh et al. reported SBM incidence rates from 2010 to 2015, as well as average APCs [22]. Due to a lack of research time, the Joinpoint regression model was unavailable for fitting incidence rate data and generating more accurate APCs data in previous research [22]. We further reported the trends in the age-standardized incidence rate of SBMs in the specific populations from 2010 to 2019 and offered a thorough analysis of how incidence rates evolved. Upon reviewing the literature in detail, we found that another article related to the trends in incidence of BMs was published in 2012 [2]; it reviewed the global incidence and prevalence of BMs using the available data from 1986 to 2016 [23,24] and found that the incidence proportion of BMs was hypothesized to have increased during the last 20 years. Between 1987 and 2006, the annual age-adjusted incidence rate of hospitalization for BMs increased from 7 to 14 individuals per 100,000 [23]. According to Tabouret et al., the use of sophisticated diagnostic imaging techniques such as magnetic resonance imaging (MRI) and advances in the standard of neuroimaging are responsible for a rise in the incidence of BMs [2,25]. Other studies likewise supported the same idea that such short-term changes in cancer epidemiological patterns are considerably more likely to be explained by modifications in clinical policy and/or practice, such as screening methods and the application of advanced diagnostic imaging [26,27,28]. Increased incidence and prevalence of solid malignancies, as well as increased physician and patient awareness of BMs, were reported as two other potential reasons for the increase in BMs incidence [23,29,30,31]. The theories presented here are presumably applicable to explain the increased incidence of melanoma, breast cancer, kidney cancer, and colorectal cancer with SBMs.

Another important finding was that the age-adjusted incidence rates of overall SBMs and lung cancer SBMs continued to decrease gradually in the last ten years (APC = −0.6 and 95% CI, −1.1 to 0; APC = −1.2 and 95% CI, −1.8 to −0.6), which contrasted with the changing patterns of SEM incidence rates, which exhibited an ascent followed by a fall. Reasons for the decreasing trend in overall SBM incidence are hard to explore due to the complex composition of the primary tumors of SBMs. However, we found that more than 80% of SBMs metastasize from the lung (Figure 1A), which indicates that the overall incidence of SBMs depends largely upon the incidence of lung cancer SBMs. So, we searched for potential causes for the declining trend in lung cancer SBM incidence. A possible explanation for this might be that among the U.S. male and female population, the incidence of lung cancer decreased gradually in recent years [32,33,34]. Tobacco epidemics in countries, socioeconomic and educational status, and the timing of diagnosis were associated with the incidence of lung cancer [35,36,37,38,39]. Based on the above analysis, we surmised that the overall SBM and lung cancer SBM age-adjusted incidence rates displayed an increasing trend in China, and central and eastern Europe among the female population and a decreasing trend in central and eastern Europe among the male population, except Norway, Finland, Spain, and France, where the incidence is supposed to be stable [33]. Limited reports are available on the recent trends in BM incidence, which hampers further analyses of the association between the trends in BMs and primary tumor incidence rates.

It is more accurate to measure progress against cancer using mortality rates rather than incidence or survival, since mortality rates are less influenced by changes in detection practices [34,40]. We examined the 10-year mortality rates in certain areas of the U.S. using the most recent data available. Before 2012, there was a noticeable increase in the incidence-based mortality rates by SBMs followed by a gradual drop. Most additional subgroup analyses revealed a similar pattern. This finding may be explained by the possibility that SBM-incidence-based mortality rates were underestimated in the early years following the initial inclusion of cases [14,41]. A quick increase in SEM mortality rates from 2010 to 2012 further proves our viewpoint. To mitigate the impact of this possible bias, it was suggested that a buffer time be eliminated from the computation of mortality rates.

Even though it is somewhat impacted by the recent fall in incidence rates, the considerable decline in SBM mortality rates indicates that we have achieved some progress in the anti-cancer process during this time. In the current therapy of BMs, tissue from resection can be utilized for genetic analyses to direct the selection of targeted therapies in the future, depending upon the type of primary tumor [42]. Minimally invasive surgery is increasingly being performed to acquire tissue for examination. Undoubtedly, BMs are now receiving more individualized care rather than being treated as a homogenous group of patients [43]. First-generation EGFR tyrosine kinase inhibitors (TKIs) (e.g., gefitinib and erlotinib) and second-generation TKIs (e.g., neratinib and dacomitinib) were shown to prolong overall survival in BMs from non-small cell lung cancer (NSCLC) with EGFR mutations [44,45]. In particular, the third-generation EGFR-TKI, Osimertinib, the first-line TKI of choice for EGFR-mutant lung cancer with BMs, was demonstrated to be more efficient [46,47]. TKI therapies were reported to be effective treatment strategies for patients with BRAF-mutant melanoma with BMs [48,49,50]. Similarly, HER2-targeted TKIs (e.g., tucatinib) significantly improved overall survival and progression-free survival of HER2+ breast cancer cases with BMs [51,52]. Additionally, in individuals with BMs from particular subtypes, ICIs demonstrated promising effectiveness [53,54,55]. Our relative survival analyses confirmed that the survival outcomes of SBMs improved. It was also obvious that the 1- to 5-year relative survival rate increased every year, but the 5-year survival rate remained extremely low. It is imperative to discover new therapeutic targets and protocols.

### Limitations

Several limitations of the present study should be discussed. It is only conceivable to make assumptions regarding the probable causes of the observed SBM trends because this study is descriptive in nature. Because there are no other published studies to which we could refer, we were unable to compare our findings with trends in the incidence and mortality of SBMs in other countries or in the U.S. prior to 2010. Our consideration of the epidemiological distinction between BMs and SBMs was constrained by the absence of extensive epidemiological data on current BM trends in the U.S. We were unable to fully comprehend the changes in trends of incidence of SBMs and mortality because individual-level environmental exposures, lifestyle-related variables, and techniques of SBM diagnosis were not recorded by the SEER program. The current study did not assess the impact of therapy on trends in incidence-based mortality that indicate long-term trends in detection, diagnosis, case identification, and related survival.

## 5. Conclusions

Between 2010 and 2019, the overall incidence of SBMs among U.S. patients declined by 0.6% per year, whereas between 2012 and 2019, the overall incidence-based mortality of SBMs decreased by 1.8% annually. There was a difference in the trends in SBM and SEM incidence and mortality rates. Since SEER cannot offer statistics to determine causality, additional research should be performed to determine whether this trend continued after 2019. These investigations might aid in the formulation of strategies for screening programs and a more effective use of regional healthcare resources.

## Figures and Tables

**Table 1:** SBM* incidence and incidence-based mortality (2010–2019): the SEER-17registry database.

Characteristic	Incidence		Incidence-Based Mortality	
	Cases, No. (%)	Rate (95% CI)	Cases, No. (%)	Rate (95% CI)
Overall	66,655 (100)	5.41 (5.37, 5.46)	57,692 (100)	4.7 (4.67, 4.74)
Age at Diagnosis/Death, y				
20–39	1181 (1.77)	0.27 (0.26, 0.29)	694 (1.2)	0.16 (0.15, 0.17)
40–59	18,283 (27.43)	3.8 (3.75, 3.86)	14,154 (24.53)	2.92 (2.88, 2.97)
60–79	39,757 (59.65)	17.56 (17.38, 17.73)	35,386 (61.34)	15.72 (15.56, 15.89)
≥80	7434 (11.15)	13.83 (13.52, 14.15)	7458 (12.93)	13.85 (13.54, 14.17)
Sex				
Male	34,821 (52.24)	6.21 (6.15, 6.28)	30,657 (53.14)	5.53 (5.47, 5.59)
Female	31,834 (47.76)	4.8 (4.75, 4.86)	27,035 (46.86)	4.07 (4.02, 4.12)
Race				
White	53,257 (79.9)	5.41 (5.36, 5.45)	46,491 (80.58)	4.73 (4.68, 4.77)
Black	7418 (11.13)	6.31 (6.16, 6.46)	6535 (11.33)	5.65 (5.51, 5.79)
Other*	5980 (8.97)	4.63 (4.52, 4.76)	4666 (8.09)	3.69 (3.58, 3.8)
Median Household Income				
<75,000	47,386 (71.09)	5.64 (5.59, 5.69)	41,445 (71.84)	4.95 (4.9, 5)
≥75,000	19,269 (28.91)	4.94 (4.87, 5.01)	16,247 (28.16)	4.19 (4.12, 4.26)
Rural–Urban Distribution				
Urban	56,598 (84.91)	5.26 (5.21, 5.3)	48,649 (84.33)	4.54 (4.5, 4.58)
Rural	10,057 (15.09)	6.6 (6.47, 6.73)	9043 (15.67)	5.91 (5.78, 6.03)
Primary Tumor Site				
Head and neck	213 (0.32)	0.02 (0.02, 0.02)	185 (0.32)	0.02 (0.01, 0.02)
Thyroid	159 (0.24)	0.01 (0.01, 0.02)	122 (0.21)	0.01 (0.01, 0.01)
Lung	53,492 (80.25)	4.34 (4.30, 4.38)	46,854 (81.21)	3.82 (3.79, 3.86)
Breast	2571 (3.86)	0.21 (0.20, 0.22)	2004 (3.47)	0.16 (0.16, 0.17)
Colorectal	977 (1.47)	0.08 (0.07, 0.08)	868 (1.5)	0.07 (0.07, 0.08)
Kidney	2054 (3.08)	0.16 (0.16, 0.17)	1733 (3)	0.14 (0.13, 0.15)
Melanoma	2812 (4.22)	0.23 (0.22, 0.24)	2231 (3.87)	0.18 (0.18, 0.19)
Liver	284 (0.43)	0.02 (0.02, 0.03)	264 (0.46)	0.02 (0.02, 0.02)
Ovarian	141 (0.21)	0.01 (0.01, 0.01)	121 (0.21)	0.01 (0.01, 0.01)
Endometrial	240 (0.36)	0.02 (0.02,0.02)	199 (0.34)	0.02 (0.01,0.02)
Prostate	406 (0.61)	0.03 (0.03, 0.04)	295 (0.51)	0.02 (0.02, 0.03)
Other	3306 (4.96)	0.27 (0.26, 0.28)	2816 (4.88)	0.23 (0.22, 0.24)
T-Stage				
1	7564 (11.35)	0.60 (0.59, 0.62)	6235 (10.81)	0.50 (0.49, 0.51)
2	14,227 (21.34)	1.15 (1.13, 1.17)	12,385 (21.47)	1.00 (0.99, 1.02)
3	12,502 (18.76)	1.00 (0.98, 1.02)	11,087 (19.22)	0.89 (0.87, 0.91)
4	17,786 (26.68)	1.43 (1.41, 1.45)	15,584 (27.01)	1.26 (1.24, 1.28)
Other	15,108 (22.67)	1.24 (1.22, 1.26)	12,877 (22.32)	1.06 (1.04, 1.07)
N-Stage				
0	15,885 (23.83)	1.29 (1.27, 1.31)	13,563 (23.51)	1.11 (1.09, 1.13)
1	7909 (11.87)	0.63 (0.62, 0.65)	6704 (11.62)	0.54 (0.52, 0.55)
2	23,366 (35.06)	1.88 (1.86, 1.91)	20,774 (36.01)	1.68 (1.66, 1.70)
3	11,395 (17.1)	0.90 (0.89, 0.92)	9855 (17.08)	0.78 (0.77, 0.80)
Other	8628 (12.94)	0.71 (0.69, 0.72)	7268 (12.6)	0.60 (0.58, 0.61)
Chemotherapy				
No	33,272 (49.92)	2.74 (2.71,2.77)	30,458 (52.79)	2.51 (2.49,2.54)
Yes	33,383 (50.08)	2.67 (2.65,2.7)	27,234 (47.21)	2.19 (2.16,2.22)
Radiotherapy				
No	22,135 (33.21)	1.82 (1.8,1.85)	19,909 (34.51)	1.64 (1.62,1.67)
Yes	44,520 (66.79)	3.59 (3.56,3.63)	37,783 (65.49)	3.06 (3.03,3.09)
Surgery				
No	62,967 (94.47)	5.12 (5.08,5.16)	54,946 (95.24)	4.48 (4.44,4.52)
Yes	3688 (5.53)	0.30 (0.29,0.31)	2746 (4.76)	0.22 (0.22,0.23)

SBM*, synchronous brain metastasis. Other* races included American Indian/AK Native and Asian/Pacific Islander.

**Table 2:** Trends in SBM incidence rates* (2010–2019): the SEER-17 registry database.

Characteristic	Overall (2010–2019)		Trends					
			1			2		
	APC (95% CI)	*p*-Value	Year	APC (95% CI)	*p*-Value	Year	APC (95% CI)	*p*-Value
Overall	−0.6 (−1.1 to 0)	<0.001						
Age at Diagnosis/Death, y								
20–39	2.8 (0.7 to 5)	0.015						
40–59	−1.9 (−3.2 to −0.5)	0.007	2010–2016	−0.8 (−2.3 to 0.7)	0.228	2016–2019	−4 (−8.1 to 0.4)	0.066
60–79	−0.5 (−1 to −0.1)	0.010						
≥80	1.3 (−0.1 to 2.6)	0.103						
Sex								
Male	−0.9 (−1.4 to −0.5)	<0.001						
Female	−0.2(−0.9 to 0.5)	0.565						
Race								
White	−0.6 (−1.2 to −0.1)	0.035						
Black	−1.2 (−2.5 to 0.3)	0.096						
Other	1.6 (0.4 to 2.9)	0.018						
Median Household Income								
<75,000	−0.4 (−0.9 to 0.1)	0.111						
≥75,000	−0.4 (−1.6 to 0.8)	0.496	2010–2015	1.4 (−0.6 to 3.3)	0.128	2015–2019	−2.6 (−5.2 to 0.1)	0.053
Rural–Urban Distribution								
Urban	−0.6 (−1.1 to −0.1)	0.033						
Rural	−0.1 (−1.2 to 1.1)	0.884						
Primary Tumor Site								
Head and neck	−1 (−4.1 to 2.1)	0.466						
Thyroid	5.1 (−6 to 17.4)	0.337						
Lung	−1.2 (−1.8 to −0.6)	0.002						
Breast	0.6 (−1.3 to 2.5)	0.508						
Colorectal	1.9 (−0.1 to 3.9)	0.064						
Kidney	0.5 (−0.2 to 1.2)	0.142						
Melanoma	2.5 (0.9 to 4.2)	0.008						
Liver	3.4 (0.4 to 6.5)	0.029						
Ovarian	0.3 (−5.4 to 6.3)	0.919						
Endometrial	0.5 (−3.5 to 4.7)	0.774						
Prostate	3.1 (−1.6 to 8.1)	0.165						
Other	4.2 (1.8 to 6.7)	0.004						
T-Stage								
1	0.7 (−0.5 to 1.8)	0.217						
2	−4.0 (−6.1 to −2)	<0.001	2010–2017	0.1 (−1.4 to 1.6)	0.898	2017–2019	−17.2 (−26.2 to −7.1)	0.008
3	−3.5 (−7.3 to 0.5)	0.084	2010–2014	3.4 (−5.4 to 13)	0.381	2014–2019	−8.7 (−14.3 to −2.7)	0.014
4	0.2 (−0.5 to 0.9)	0.500						
Other	2.9 (0.5 to 5.4)	0.016	2010–2016	−1.7 (−4.3 to 0.9)	0.147	2016–2019	13.0 (4.6 to 22.1)	0.010
N-Stage								
0	−1.6 (−2.6 to −0.6)	0.001	2010–2014	1 (−1.2 to 3.2)	0.305	2014–2019	−3.6 (−5.1 to −2.1)	0.002
1	−0.5 (−1.4 to 0.3)	0.195						
2	−3.4 (−5 to −1.7)	0.002						
3	2.0 (0.8 to 3.2)	0.004						
Other	5.7 (2.5 to 8.8)	<0.001	2010–2015	−3.7 (−8.1 to 0.9)	0.093	2015–2019	18.6 (11 to 26.7)	0.001
Chemotherapy								
No	−0.6 (−1.7, 0.5)	0.219						
Yes	−0.4 (−1.3, 0.5)	0.388	2010–2014	1.6 (−0.4, 3.7)	0.099	2014–2019	−2 (−3.4, −0.6)	0.016
Radiotherapy								
No	0.9 (0, 1.7)	0.04						
Yes	−1.3 (−2.1, −0.4)	0.008						
Surgery								
No	−0.4 (−1, 0.1)	0.127						
Yes	−2.7 (−3.9, −1.4)	0.001						

rates*, calculated as number of cases per 100,000 person-years and age-adjusted rates were standardized to U.S. 2000 population. Joinpoint regression was used to identify each segment.

**Table 3:** Trends in SBM-incidence-based mortality rates* (2010–2019): the SEER-17 registry database.

Characteristic	Overall (2010–2019)		Trends					
			1			2		
	APC (95% CI)	*p*-Value	Year	APC (95% CI)	*p*-Value	Year	APC (95% CI)	*p*-Value
Over	4.3 (2.8 to 5.8)	<0.001	2010–2012	28.6 (19 to 38.9)	<0.001	2012–2019	−1.8 (−2.8 to −0.8)	0.006
Age at Diagnosis/Death, y								
20–39	9.2 (0.9 to 18.3)	0.030	2010–2012	49.9 (−2.1 to 129.6)	0.058	2012–2019	−0.2 (−5.8 to 5.6)	0.921
40–59	37.1 (20 to 56.5)	0.001	2010–2012	37.1 (20 to 56.5)	0.002	2012–2019	−3.6 (−5.3 to −1.9)	0.003
60–78	26.6 (18.8 to 34.9)	<0.001	2010–2012	26.6 (18.8 to 34.9)	<0.001	2012–2019	−1.7 (−2.5 to −0.9)	0.003
≥80	4.9 (2.2 to 7.8)	<0.001	2010–2012	21.5 (5.4 to 40.2)	0.017	2012–2019	0.6 (−1.3 to 2.6)	0.432
Sex								
Male	3.6 (2.1 to 5.1)	<0.001	2010–2012	27.2 (17.6 to 37.5)	0.001	2012–2019	−2.3 (−3.3 to −1.3)	0.002
Female	5.0 (3.1 to 7)	<0.001	2010–2012	30.4 (17.9 to 44.1)	0.001	2012–2019	−1.3 (−2.6 to 0.1)	0.056
Race								
White	4.1 (2.4 to 5.9)	<0.001	2010–2012	28.6 (17.6 to 40.8)	0.001	2012–2019	−2.0 (−3.1 to −0.8)	0.008
Black	3.6 (1.7 to 5.5)	<0.001	2010–2012	25.0 (13.3 to 37.8)	0.002	2012–2019	−1.8 (−3.1 to −0.6)	0.015
Other	7.5 (5.1 to 10.1)	<0.001	2010–2012	35.8 (20 to 53.8)	0.001	2012–2019	0.6 (−1 to 2.3)	0.391
Median Household Income								
<75,000	4.2 (2.4 to 6)	<0.001	2010–2012	27.3 (16.3 to 39.5)	0.001	2012–2019	−1.6 (−2.8 to −0.4)	0.019
≥75,000	5.1 (2.2 to 8.1)	0.001	2010–2012	31.2 (12.8 to 52.7)	0.006	2012–2019	−1.3 (−3.3 to 0.7)	0.149
Rural–Urban Distribution								
Urban	4.2 (2.7 to 5.8)	<0.001	2010–2012	28.5 (18.7 to 39.1)	<0.001	2012–2019	−1.9 (−2.9 to −0.8)	0.006
Rural	4.9 (1.7 to 8.2)	0.002	2010–2012	30.2 (10.3 to 53.7)	0.009	2012–2019	−1.4 (−3.5 to 0.9)	0.174
Primary Tumor Site								
Head and neck	3.9 (−1.7 to 9.7)	0.148						
Thyroid	-	-						
Lung	3.6 (2.1 to 5)	<0.001	2010–2012	28.4 (19.2 to 38.2)	<0.001	2012–2019	−2.6 (−3.6 to −1.6)	0.001
Breast	9.8 (3.1 to 17)	0.004	2010–2012	44.8 (3.3 to 103)	0.037	2012–2019	1.4 (−3 to 6.1)	0.454
Colorectal	5.7 (2.3 to 9.3)	0.005						
Kidney	5.6 (−0.3 to 11.8)	0.064	2010–2012	30.1 (−4.1 to 76.7)	0.077	2012–2019	−0.6 (−4.6 to 3.6)	0.728
Melanoma	4.1 (0 to 8.4)	0.048						
Liver	4.4 (−1.7 to 10.8)	0.139						
Ovarian	0.7 (−9.2 to 11.6)	0.885						
Endometrial	1.9 (−4.9 to 9.1)	0.554						
Prostate	17.7 (5.8 to 30.9)	0.003	2010–2012	93.4 (9.4 to 242)	0.031	2012–2019	2.1 (−5.4 to 10.2)	0.512
Other	7.9 (2.2 to 13.9)	0.006	2010–2012	27 (−4.8 to 69.6)	0.086	2012–2019	3 (−0.9 to 7)	0.109
T-Stage								
1	6.5 (2.7 to 10.5)	0.001	2010–2012	33.6 (9.9 to 62.3)	0.012	2012–2019	−0.2 (−2.7 to 2.5)	0.880
2	3.8 (−2.9 to 10.9)	0.275	2010–2012	36.2 (−4.6 to 94.3)	0.076	2012–2019	−4 (−8.4 to 0.7)	0.080
3	2.6 (−2.5 to 8)	0.327	2010–2012	38.8 (5.5 to 82.6)	0.028	2012–2019	−5.9 (−9.3 to −2.4)	0.008
4	4.2 (2.7 to 5.7)	<0.001	2010–2012	25.0 (15.5 to 35.3)	0.001	2012–2019	−1.1 (−2.1 to 0)	0.044
Other	3.9 (0.7 to 7.1)	0.022						
N-Stage								
0	4.7 (1.5 to 8)	0.003	2010–2012	35.5 (15 to 59.7)	0.005	2012–2019	−2.7 (−4.8 to −0.6)	0.023
1	4.9 (2.7 to 7.2)	<0.001	2010–2012	33.4 (19.1 to 49.5)	0.001	2012–2019	−2.0 (−3.5 to −0.5)	0.017
2	1.9 (−1.1 to 5)	0.225	2010–2012	30.6 (11.1 to 53.6)	0.008	2012–2019	−5.1 (−7.1 to −3)	0.002
3	6.5 (2.9 to 10.1)	<0.001	2010–2012	30.5 (9 to 56.3)	0.013	2012–2019	0.4 (−1.9 to 2.9)	0.657
Other	6.1 (2.9 to9.4)	0.002						
Chemotherapy								
No	1.6 (−0.3, 3.6)	0.094	2010–2012	14.4 (3.3, 26.7)	0.02	2012–2019	−1.7 (−3.1, −0.4)	0.022
Yes	8.4 (5.5, 11.3)	<0.001	2010–2012	54.1 (33.6, 77.8)	0.001	2012–2019	−2 (−3.8, −0.1)	0.042
Radiotherapy								
No	3.2 (1.2, 5.4)	0.002	2010–2012	14.1 (2.4, 27.2)	0.026	2012–2019	0.3 (−1.1, 1.8)	0.591
Yes	4.9 (3.2, 6.7)	<0.001	2010–2012	38 (26.1, 50.9)	<0.001	2012–2019	−3 (−4.1, −1.8)	0.001
Surgery								
No	4.1 (2.7, 5.6)	<0.001	2010–2012	27.8 (18.5, 37.9)	<0.001	2012–2019	−1.8 (−2.8, −0.8)	0.006
Yes	7.4 (3.9, 10.9)	<0.001	2010–2012	46.9 (23.6, 74.6)	0.002	2012–2019	−1.8 (−4.1, 0.5)	0.094

rates*, calculated as number of deaths per 100,000 person-years and age-adjusted rates were standardized to U.S. 2000 population. Joinpoint regression was used to identify each segment.

## Data Availability

The data presented in this study are openly available in: https://seer.cancer.gov assessed on 30 May 2022.

## Supplementary Materials

The following supporting information can be downloaded at: https://www.mdpi.com/article/10.3390/curroncol29110660/s1.
