# Supplementary material for: Recent Trends in Synchronous Brain Metastasis Incidence and Mortality in the United States: Ten-Year Multicenter Experience
Source: Curr Oncol. 2022 Nov 2;29(11):8374–89. doi: 10.3390/curroncol29110660 (PMC9689090; doi:10.3390/curroncol29110660)
Supplement: Supplementary file 1 [file curroncol-29-00660-s001.zip › curroncol-1996790-supplementary.pdf]

Table S1. Top 20 SEER Files in the Rate Session.

| Types of Databases | Registries | Submission Date | Time Periods |
|---|---|---|---|
| Incidence-SEER Research Data | 8 | November 2021 | 1975-2019 |
| Incidence-SEER Research Data | 12 | November 2021 | 1992-2019 |
| Incidence-SEER Research Data | 12 (excl AK) | November 2021 | 1992-2019 |
| Incidence-SEER Research Data | 17 | November 2021 | 2000-2019 |
| Incidence-SEER Research Data | 17 (excl AK) | November 2021 | 2000-2019 |
| Incidence-SEER Research Limited-Field Data | 22 | November 2021 | 2000-2019 |
| Incidence-Based Mortality-SEER Research Data | 8 | November 2021 | 1975-2019 |
| Incidence-Based Mortality-SEER Research Data | 12 | November 2021 | 1992-2019 |
| Incidence-Based Mortality-SEER Research Data | 17 | November 2021 | 2000-2019 |
| Incidence-SEER Research Data with Delay Adjustment | 8 | November 2021 | 1975-2019 |
| Incidence-SEER Research Data with Delay Adjustment | 12 | November 2021 | 1992-2019 |
| Incidence-SEER Research Data with Delay Adjustment | 17 | November 2021 | 2000-2019 |
| Incidence-SEER Research Limited-Field Data with Delay-Adjustment | 22 | November 2021 | 2000-2019 |
| Incidence-SEER Research Plus Data | 8 | November 2021 | 1975-2019 |
| Incidence-SEER Research Plus Data | 12 | November 2021 | 1992-2019 |
| Incidence-SEER Research Plus Data | 17 | November 2021 | 2000-2019 |
| Incidence-SEER Research Plus Limited-Field Data | 22 | November 2021 | 2000-2019 |
| Incidence-Based Mortality-SEER Research Plus Data | 8 | November 2021 | 1975-2019 |
| Incidence-Based Mortality-SEER Research Plus Data | 12 | November 2021 | 1992-2019 |
| Incidence-Based Mortality-SEER Research Plus Data | 17 | November 2021 | 2000-2019 |

Table S2. Topography or morphology codes of primary tumor.

| Primary tumor site | ICD-O-3* Topography or Morphology codes |
|---|---|
| Head & Neck | C0, C1, C2, C3, C4, C5, C6, C7, C8, C9, C10, C11, C12, C13, C14, C76.0 |
| Thyroid | C73.9 |
| Lung | C34 |
| Breast | C50 |
| Colorectal | C18, C19, C20, C21 |
| Kidney | C64.9 |
| Melanoma | 872, 873, 874, 875, 876, 877, 878, 879 |
| Liver | C22 |
| Ovarian | C56 |
| Endometrial | C54.1 |
| Prostate | C61.9 |

ICD-O-3*, International Classification of Diseases for Oncology 3rd Edition, 2000.

Table S3. SBMs* Incidence Rates* and Incidence-Based Mortality Rates*.

| Group (year) | Incidence Male No. of Cases | Rate | Female No. of Cases | Rate | M/F* Rate Ratios (95% CI) | Incidence-based mortality Male No. of Cases | Rate | Female No. of Cases | Rate | M/F Rate Ratios (95% CI) |
|---|---|---|---|---|---|---|---|---|---|---|
| **Age** | | | | | | | | | | |
| 20-39 | 582 | 0.26(0.24,0.28) | 599 | 0.28(0.26,0.3) | 0.96(0.87,1.06) | 363 | 0.16(0.15,0.18) | 331 | 0.16(0.14,0.17) | 1.02(0.90,1.16) |
| 40-59 | 9,159 | 3.88(3.8,3.96) | 9,124 | 3.74(3.66,3.82) | 1.02(0.99,1.04) | 7,286 | 3.06(2.99,3.13) | 6,868 | 2.79(2.73,2.86) | 1.05(1.02,1.08) |
| 60-79 | 21,230 | 20.42(20.14,20.7) | 18,527 | 15.15(14.93,15.37) | 1.16(1.14,1.18) | 19,134 | 18.53(18.26,18.8) | 16,252 | 13.36(13.16,13.57) | 1.18(1.16,1.20) |
| ⩾80 | 3,850 | 19.12(18.52,19.73) | 3,584 | 10.7(10.35,11.06) | 1.38(1.33,1.44) | 3,874 | 19.25(18.65,19.87) | 3,584 | 10.66(10.31,11.02) | 1.39(1.34,1.45) |
| **Race** | | | | | | | | | | |
| White | 27,802 | 6.13(6.06,6.21) | 25,455 | 4.85(4.79,4.91) | 1.13(1.12,1.15) | 24,631 | 5.48(5.41,5.55) | 21,860 | 4.14(4.09,4.2) | 1.16(1.14,1.18) |
| Black | 3,934 | 7.84(7.58,8.1) | 3,484 | 5.23(5.06,5.41) | 1.24(1.19,1.29) | 3,525 | 7.18(6.93,7.43) | 3,010 | 4.57(4.41,4.74) | 1.27(1.22,1.33) |
| Other* | 3,085 | 5.47(5.27,5.67) | 2,895 | 4.02(3.88,4.17) | 1.18(1.13,1.23) | 2,501 | 4.54(4.72,2501) | 2,165 | 3.05(3.19,2165) | 1.23(1.17,1.29) |
| **MHHI** | | | | | | | | | | |
| <75000 | 25,226 | 6.58(6.5,6.67) | 22,160 | 4.91(4.85,4.98) | 1.17(1.15,1.19) | 22,372 | 5.9(5.82,5.97) | 19,073 | 4.22(4.16,4.28) | 1.19(1.17,1.21) |
| ⩾75000 | 9,595 | 5.44(5.33,5.55) | 9,674 | 4.58(4.49,4.67) | 1.10(1.07,1.13) | 8,285 | 4.76(4.65,4.86) | 7,962 | 3.76(3.68,3.85) | 1.14(1.11,1.17) |
| **Rural-Urban Distribution** | | | | | | | | | | |
| Urban | 29,259 | 6(5.93,6.07) | 27,339 | 4.7(4.64,4.75) | 1.14(1.12,1.16) | 25,575 | 5.31(5.24,5.37) | 23,074 | 3.96(3.91,4.01) | 1.17(1.15,1.19) |
| Rural | 5,562 | 7.76(7.55,7.97) | 4,495 | 5.65(5.48,5.82) | 1.18(1.14,1.22) | 5,082 | 7.1(6.91,7.3) | 3,961 | 4.93(4.78,5.09) | 1.20(1.16,1.25) |

SBMs*, synchronous brain metastases; MHHI*, median household income; Rates*, calculated as number of cases per 100,000 person-years and age-adjusted to the 2000 U.S. standard population; M/F*, Male/Female. Other* race included American Indian/AK Native and Asian/Pacific Islander.

Table S4. Relative Survival (Percent) According to Year of Diagnosis.

SBMs* Relative Survival (Percent) According to Year of Diagnosis

| Survival time | 2010 | 2011 | 2012 | 2013 | 2014 | 2015 | 2016 | 2017 | 2018 |
|---|---|---|---|---|---|---|---|---|---|
| 1-year | 23.38 | 25.16 | 25.87 | 26.64 | 27.19 | 29.14 | 32.02 | 31.75 | 34.45 |
| 2-year | 10.28 | 11.88 | 12.19 | 13.23 | 12.98 | 15.66 | 18.32 | 18.96 | |
| 3-year | 5.93 | 7.15 | 7.27 | 7.99 | 8.88 | 10.84 | 12.33 | | |
| 4-year | 4.22 | 5.1 | 5.47 | 5.76 | 6.38 | 8.12 | | | |
| 5-year | 3.4 | 4 | 4.29 | 4.7 | 5.24 | | | | |
| 6-year | 2.83 | 3.07 | 3.65 | 3.97 | | | | | |
| 7-year | 2.57 | 2.71 | 3.34 | | | | | | |
| 8-year | 2.3 | 2.4 | | | | | | | |
| 9-year | 2.13 | | | | | | | | |

Lung cancer SBMs* Relative Survival (Percent) According to Year of Diagnosis

| Survival time | 2010 | 2011 | 2012 | 2013 | 2014 | 2015 | 2016 | 2017 | 2018 |
|---|---|---|---|---|---|---|---|---|---|
| 1-year | 23.18 | 25.63 | 25.5 | 26.49 | 27.21 | 29.37 | 31.94 | 31.46 | 34.44 |
| 2-year | 9.91 | 11.77 | 11.6 | 12.56 | 12.45 | 15.54 | 18.06 | 18.65 | |
| 3-year | 5.49 | 6.9 | 6.64 | 7.4 | 8.19 | 10.34 | 11.89 | | |
| 4-year | 3.79 | 4.75 | 4.75 | 5.17 | 5.75 | 7.46 | | | |
| 5-year | 2.95 | 3.56 | 3.51 | 4.07 | 4.56 | | | | |
| 6-year | 2.39 | 2.7 | 2.91 | 3.33 | | | | | |
| 7-year | 2.14 | 2.37 | 2.65 | | | | | | |
| 8-year | 1.87 | 2.02 | | | | | | | |
| 9-year | 1.66 | | | | | | | | |

Breast cancer SBMs* Relative Survival (Percent) According to Year of Diagnosis

| Survival | 2010 | 2011 | 2012 | 2013 | 2014 | 2015 | 2016 | 2017 | 2018 |
|---|---|---|---|---|---|---|---|---|---|

| time | | | | | | | | | |
|---|---|---|---|---|---|---|---|---|---|
| 1-year | 46.11 | 44.07 | 49.8 | 47.43 | 47.11 | 48.89 | 45.33 | 53.37 | 48.16 |
| 2-year | 27.84 | 30.4 | 29.82 | 29.86 | 24.29 | 30.32 | 32.16 | 36.2 | |
| 3-year | 21.78 | 21.43 | 18.57 | 21.96 | 18.21 | 23 | 23.07 | | |
| 4-year | 15.6 | 14.84 | 15.45 | 13.65 | 13.03 | 15.84 | | | |
| 5-year | 13.4 | 11.72 | 10.16 | 9.89 | 12.48 | | | | |
| 6-year | 9.41 | 8.29 | 8.21 | 8.26 | | | | | |
| 7-year | 8.88 | 7.26 | 6.76 | | | | | | |
| 8-year | 6.59 | 6.24 | | | | | | | |
| 9-year | 6.59 | | | | | | | | |

Melanoma SBMs* Relative Survivala (Percent) According to Year of Diagnosis

| Survival time | 2010 | 2011 | 2012 | 2013 | 2014 | 2015 | 2016 | 2017 | 2018 |
|---|---|---|---|---|---|---|---|---|---|
| 1-year | 21.66 | 22.4 | 30.89 | 30.12 | 28.94 | 33.61 | 37.1 | 40.82 | 38.47 |
| 2-year | 11.52 | 14.38 | 18.89 | 17.08 | 19.98 | 18.47 | 25.55 | 28.97 | |
| 3-year | 6.46 | 13.4 | 14.66 | 12.72 | 17.86 | 15.78 | 20.92 | | |
| 4-year | 5.86 | 11.89 | 12.63 | 11.14 | 14.76 | 15.42 | | | |
| 5-year | 5.86 | 10.84 | 11.41 | 10.07 | 14.03 | | | | |
| 6-year | 5.86 | 9.11 | 9.2 | 10.07 | | | | | |
| 7-year | 5.86 | 8 | 7.78 | | | | | | |
| 8-year | 5.86 | 8 | | | | | | | |
| 9-year | 5.86 | | | | | | | | |

SBMs*, synchronous brain metastases.

Table S5. SBMs* Incidence Rates* for Histologic Type, T-Stage, and N-Stage.

| SBMs incidence by primary tumor site. | | | | | | | | | | | | |
|---|---|---|---|---|---|---|---|---|---|---|---|---|
| | Total | | Lung | | Breast | | Kidney | | Melanoma | | Colorectal | |
| Year of diagnosis | No. of Cases | Rate | No. of Cases | Rate | No. of Cases | Rate | No. of Cases | Rate | No. of Cases | Rate | No. of Cases | Rate |
| 2010 | 6,091 | 9.93 | 4,988 | 8.13 | 235 | 0.38 | 176 | 0.28 | 230 | 0.38 | 88 | 0.14 |
| 2011 | 6,131 | 9.76 | 4,968 | 7.92 | 249 | 0.39 | 193 | 0.29 | 251 | 0.4 | 87 | 0.14 |
| 2012 | 6,418 | 10 | 5,288 | 8.22 | 216 | 0.34 | 197 | 0.3 | 217 | 0.35 | 93 | 0.14 |
| 2013 | 6,510 | 9.87 | 5,285 | 8 | 265 | 0.4 | 202 | 0.3 | 260 | 0.41 | 82 | 0.12 |
| 2014 | 6,755 | 9.96 | 5,463 | 8.05 | 254 | 0.38 | 203 | 0.29 | 306 | 0.46 | 102 | 0.15 |
| 2015 | 6,745 | 9.76 | 5,410 | 7.82 | 272 | 0.4 | 204 | 0.29 | 295 | 0.43 | 95 | 0.14 |
| 2016 | 6,767 | 9.54 | 5,357 | 7.5 | 230 | 0.34 | 216 | 0.3 | 292 | 0.43 | 99 | 0.14 |
| 2017 | 7,246 | 10.02 | 5,751 | 7.89 | 292 | 0.42 | 221 | 0.31 | 295 | 0.42 | 98 | 0.14 |
| 2018 | 6,927 | 9.35 | 5,476 | 7.34 | 267 | 0.37 | 213 | 0.29 | 318 | 0.45 | 110 | 0.16 |
| 2019 | 7,065 | 9.39 | 5,506 | 7.24 | 291 | 0.41 | 229 | 0.3 | 348 | 0.49 | 123 | 0.17 |
| SBMs incidence by T-Stage. | | | | | | | | | | | | |
| | T1 | | T2 | | T3 | | T4 | | Other | | | |
| Year of diagnosis | No. of Cases | Rate | No. of Cases | Rate | No. of Cases | Rate | No. of Cases | Rate | No. of Cases | Rate | | |
| 2010 | 630 | 1.03 | 1334 | 2.18 | 1147 | 1.85 | 1612 | 2.60 | 1368 | 2.26 | | |
| 2011 | 677 | 1.07 | 1322 | 2.12 | 1204 | 1.90 | 1580 | 2.50 | 1348 | 2.17 | | |
| 2012 | 688 | 1.06 | 1462 | 2.30 | 1337 | 2.08 | 1626 | 2.51 | 1305 | 2.04 | | |
| 2013 | 694 | 1.04 | 1403 | 2.12 | 1307 | 1.97 | 1767 | 2.68 | 1339 | 2.05 | | |
| 2014 | 720 | 1.07 | 1467 | 2.16 | 1416 | 2.09 | 1741 | 2.55 | 1411 | 2.08 | | |
| 2015 | 736 | 1.07 | 1485 | 2.15 | 1415 | 2.02 | 1765 | 2.55 | 1344 | 1.96 | | |
| 2016 | 807 | 1.12 | 1569 | 2.22 | 1203 | 1.68 | 1776 | 2.50 | 1412 | 2.03 | | |
| 2017 | 873 | 1.21 | 1652 | 2.29 | 1317 | 1.81 | 1853 | 2.55 | 1551 | 2.18 | | |
| 2018 | 820 | 1.09 | 1225 | 1.65 | 979 | 1.34 | 1916 | 2.58 | 1987 | 2.70 | | |
| 2019 | 786 | 1.04 | 1175 | 1.57 | 1044 | 1.38 | 2017 | 2.67 | 2043 | 2.75 | | |

| SBMs incidence by N-Stage. | | | | | | | | | | | | |
|---|---|---|---|---|---|---|---|---|---|---|---|---|
| | N0 | | N1 | | N2 | | N3 | | Other | | | |
| Year of diagnosis | No. of Cases | Rate | No. of Cases | Rate | No. of Cases | Rate | No. of Cases | Rate | No. of Cases | Rate | | |
| 2010 | 1,434 | 2.38 | 699 | 1.13 | 2,300 | 3.72 | 904 | 1.45 | 754 | 1.25 | | |
| 2011 | 1,522 | 2.44 | 729 | 1.15 | 2,298 | 3.65 | 900 | 1.42 | 682 | 1.1 | | |
| 2012 | 1,547 | 2.44 | 752 | 1.18 | 2,410 | 3.73 | 1,017 | 1.57 | 692 | 1.09 | | |
| 2013 | 1,580 | 2.41 | 766 | 1.17 | 2,427 | 3.67 | 1,072 | 1.59 | 665 | 1.03 | | |
| 2014 | 1,696 | 2.51 | 790 | 1.17 | 2,436 | 3.58 | 1,145 | 1.66 | 688 | 1.03 | | |
| 2015 | 1,656 | 2.44 | 810 | 1.17 | 2,492 | 3.58 | 1,163 | 1.65 | 624 | 0.92 | | |
| 2016 | 1,581 | 2.24 | 775 | 1.10 | 2,302 | 3.23 | 1,234 | 1.71 | 875 | 1.26 | | |
| 2017 | 1,599 | 2.24 | 827 | 1.15 | 2,478 | 3.42 | 1,327 | 1.81 | 1,015 | 1.43 | | |
| 2018 | 1,600 | 2.17 | 779 | 1.05 | 2,014 | 2.71 | 1,211 | 1.62 | 1,323 | 1.8 | | |
| 2019 | 1,538 | 2.06 | 850 | 1.14 | 2,077 | 2.73 | 1,290 | 1.70 | 1,310 | 1.76 | | |

SBMs*, synchronous brain metastases.

Rates* were calculated as number of cases per 100,000 person-years and age-adjusted to the 2000 U.S. standard population.

Table S6. SBMs* Incidence-Based Mortality Rates* for Histologic Type, T-Stage, and N-Stage.

SBMs incidence-based mortality by primary tumor site.

| | Total | | Lung | | Breast | | Kidney | | Melanoma | | Colorectal | |
|---|---|---|---|---|---|---|---|---|---|---|---|---|
| Year of diagnosis | No. of Cases | Rate | No. of Cases | Rate | No. of Cases | Rate | No. of Cases | Rate | No. of Cases | Rate | No. of Cases | Rate |
| 2010 | 3,259 | 5.4 | 2,717 | 4.51 | 80 | 0.13 | 95 | 0.15 | 118 | 0.2 | 49 | 0.08 |
| 2011 | 5,163 | 8.28 | 4,259 | 6.84 | 170 | 0.27 | 152 | 0.24 | 203 | 0.33 | 76 | 0.12 |
| 2012 | 5,751 | 8.96 | 4,763 | 7.41 | 168 | 0.27 | 175 | 0.27 | 192 | 0.31 | 79 | 0.12 |
| 2013 | 5,942 | 9.05 | 4,936 | 7.52 | 190 | 0.29 | 158 | 0.24 | 207 | 0.33 | 74 | 0.11 |
| 2014 | 6,201 | 9.17 | 5,026 | 7.44 | 216 | 0.32 | 194 | 0.28 | 270 | 0.41 | 94 | 0.13 |
| 2015 | 6,350 | 9.18 | 5,186 | 7.49 | 257 | 0.38 | 191 | 0.27 | 226 | 0.34 | 86 | 0.12 |
| 2016 | 6,162 | 8.73 | 4,968 | 7.01 | 213 | 0.31 | 179 | 0.25 | 253 | 0.37 | 83 | 0.12 |
| 2017 | 6,386 | 8.82 | 5,082 | 6.97 | 230 | 0.33 | 211 | 0.3 | 238 | 0.34 | 102 | 0.15 |
| 2018 | 6,334 | 8.53 | 5,063 | 6.77 | 225 | 0.31 | 191 | 0.26 | 259 | 0.37 | 95 | 0.13 |
| 2019 | 6,144 | 8.16 | 4,854 | 6.39 | 255 | 0.35 | 187 | 0.25 | 265 | 0.36 | 130 | 0.18 |

SBMs incidence-based mortality by T-Stage.

| | T1 | | T2 | | T3 | | T4 | | Other | | | |
|---|---|---|---|---|---|---|---|---|---|---|---|---|
| Year of diagnosis | No. of Cases | Rate | No. of Cases | Rate | No. of Cases | Rate | No. of Cases | Rate | No. of Cases | Rate | | |
| 2010 | 288 | 0.49 | 667 | 1.12 | 636 | 1.04 | 918 | 1.50 | 750 | 1.25 | | |
| 2011 | 533 | 0.86 | 1104 | 1.77 | 982 | 1.56 | 1349 | 2.15 | 1,195 | 1.94 | | |
| 2012 | 569 | 0.88 | 1269 | 2.00 | 1181 | 1.83 | 1501 | 2.32 | 1,231 | 1.92 | | |
| 2013 | 620 | 0.94 | 1326 | 2.03 | 1200 | 1.83 | 1597 | 2.41 | 1,199 | 1.84 | | |
| 2014 | 652 | 0.97 | 1289 | 1.92 | 1321 | 1.95 | 1644 | 2.42 | 1,295 | 1.91 | | |
| 2015 | 638 | 0.93 | 1411 | 2.04 | 1342 | 1.93 | 1705 | 2.46 | 1,254 | 1.83 | | |
| 2016 | 647 | 0.91 | 1390 | 1.98 | 1169 | 1.65 | 1668 | 2.34 | 1,288 | 1.84 | | |
| 2017 | 745 | 1.04 | 1459 | 2.03 | 1188 | 1.61 | 1640 | 2.25 | 1,354 | 1.9 | | |
| 2018 | 734 | 0.97 | 1287 | 1.73 | 1020 | 1.37 | 1723 | 2.31 | 1,570 | 2.14 | | |
| 2019 | 690 | 0.90 | 1064 | 1.42 | 929 | 1.24 | 1720 | 2.28 | 1,741 | 2.34 | | |

| SBMs incidence-based mortality by N-Stage. | | | | | | | | | | | | |
|---|---|---|---|---|---|---|---|---|---|---|---|---|
| | N0 | | N1 | | N2 | | N3 | | Other | | | |
| Year of diagnosis | No. of Cases | Rate | No. of Cases | Rate | No. of Cases | Rate | No. of Cases | Rate | No. of Cases | Rate | | |
| 2010 | 700 | 1.18 | 346 | 0.57 | 1268 | 2.09 | 506 | 0.82 | 439 | 0.74 | | |
| 2011 | 1205 | 1.96 | 604 | 0.96 | 1965 | 3.14 | 782 | 1.25 | 607 | 0.98 | | |
| 2012 | 1350 | 2.13 | 652 | 1.01 | 2183 | 3.37 | 920 | 1.42 | 646 | 1.02 | | |
| 2013 | 1392 | 2.13 | 684 | 1.05 | 2313 | 3.52 | 934 | 1.39 | 619 | 0.96 | | |
| 2014 | 1552 | 2.32 | 719 | 1.06 | 2290 | 3.38 | 995 | 1.45 | 645 | 0.97 | | |
| 2015 | 1515 | 2.22 | 738 | 1.07 | 2398 | 3.44 | 1110 | 1.58 | 589 | 0.87 | | |
| 2016 | 1430 | 2.05 | 730 | 1.03 | 2184 | 3.07 | 1099 | 1.53 | 719 | 1.03 | | |
| 2017 | 1482 | 2.08 | 698 | 0.97 | 2185 | 3.00 | 1170 | 1.58 | 851 | 1.2 | | |
| 2018 | 1441 | 1.96 | 686 | 0.93 | 2039 | 2.73 | 1121 | 1.48 | 1,047 | 1.42 | | |
| 2019 | 1378 | 1.84 | 729 | 0.97 | 1831 | 2.42 | 1100 | 1.46 | 1,106 | 1.48 | | |

SBMs*, synchronous brain metastases.

Rates* were calculated as number of cases per 100,000 person-years and age-adjusted to the 2000 U.S. standard population.

Table S7. SBMs* Incidence Rate* by Sex and Race.

| Year of diagnosis | Sex | | | | Race | | | | | |
|---|---|---|---|---|---|---|---|---|---|---|
| | Male | | Female | | White | | Black | | Other* | |
| | No. cases | Rate | No. cases | Rate | No. cases | Rate | No. cases | Rate | No. cases | Rate |
| 2010 | 3,206 | 11.41 | 2,885 | 8.76 | 4,911 | 10.03 | 719 | 12.08 | 461 | 7.06 |
| 2011 | 3,235 | 11.31 | 2,896 | 8.57 | 4,994 | 10 | 671 | 10.97 | 466 | 6.9 |
| 2012 | 3,416 | 11.61 | 3,002 | 8.74 | 5,177 | 10.17 | 745 | 11.65 | 496 | 7.14 |
| 2013 | 3,365 | 11.08 | 3,145 | 8.94 | 5,261 | 10.07 | 657 | 10.12 | 592 | 8.13 |
| 2014 | 3,548 | 11.33 | 3,207 | 8.87 | 5,516 | 10.31 | 696 | 10.4 | 543 | 7.01 |
| 2015 | 3,505 | 11 | 3,240 | 8.79 | 5,373 | 9.9 | 750 | 10.77 | 622 | 7.71 |
| 2016 | 3,557 | 10.77 | 3,210 | 8.55 | 5,355 | 9.68 | 778 | 10.67 | 634 | 7.62 |
| 2017 | 3,699 | 11.1 | 3,547 | 9.17 | 5,709 | 10.12 | 836 | 11.47 | 701 | 8.13 |
| 2018 | 3,657 | 10.67 | 3,270 | 8.3 | 5,437 | 9.43 | 753 | 9.95 | 737 | 8.25 |
| 2019 | 3,633 | 10.43 | 3,432 | 8.58 | 5,524 | 9.51 | 813 | 10.4 | 728 | 7.78 |

SBMs*, synchronous brain metastases.

Rates* were calculated as number of cases per 100,000 person-years and age-adjusted to the 2000 U.S. standard population.

Other* race included American Indian/AK Native and Asian/Pacific Islander.

Table S8. SBMs* Incidence Rate* according to Age at Diagnosis.

| Year of diagnosis | Age at Diagnosis | | | | | | | |
|---|---|---|---|---|---|---|---|---|
| | 20-39 | | 40-59 | | 60-79 | | ≥80 | |
| | No. cases | Rate | No. cases | Rate | No. cases | Rate | No. cases | Rate |
| 2010 | 102 | 0.48 | 1,885 | 7.58 | 3,441 | 31.29 | 663 | 24.35 |
| 2011 | 95 | 0.45 | 1,830 | 7.3 | 3,553 | 31.19 | 653 | 23.62 |
| 2012 | 99 | 0.47 | 1,888 | 7.44 | 3,732 | 31.79 | 699 | 24.98 |
| 2013 | 111 | 0.52 | 1,923 | 7.51 | 3,773 | 30.82 | 703 | 25.14 |
| 2014 | 122 | 0.57 | 1,941 | 7.43 | 3,943 | 31.04 | 749 | 26.41 |
| 2015 | 124 | 0.57 | 1,851 | 7.1 | 3,978 | 30.3 | 792 | 27.61 |
| 2016 | 142 | 0.63 | 1,857 | 7.14 | 4,064 | 29.79 | 704 | 24.09 |
| 2017 | 126 | 0.56 | 1,827 | 7.04 | 4,436 | 31.55 | 857 | 28.86 |
| 2018 | 116 | 0.51 | 1,650 | 6.45 | 4,361 | 29.88 | 800 | 26.2 |
| 2019 | 144 | 0.62 | 1,631 | 6.44 | 4,476 | 29.88 | 814 | 26.29 |

SBMs*, synchronous brain metastases.

Rates* were calculated as number of cases per 100,000 person-years and age-adjusted to the 2000 U.S. standard population.

Table S9. SBMs* Incidence Rate* During 2010-2019 According to Median Household Income and Area Distribution.

| Year of diagnosis | Median Household Income | | | | Rural-Urban Distribution | | | |
|---|---|---|---|---|---|---|---|---|
| | <75000 | | ≥75000 | | Urban | | Rural | |
| | No. cases | Rate | No. cases | Rate | No. cases | Rate | No. cases | Rate |
| 2010 | 4,517 | 10.3 | 1,574 | 9.01 | 5,184 | 9.65 | 907 | 12.1 |
| 2011 | 4,627 | 10.17 | 1,504 | 8.73 | 5,165 | 9.39 | 966 | 12.7 |
| 2012 | 4,926 | 10.31 | 1,492 | 9.09 | 5,430 | 9.61 | 988 | 12.96 |
| 2013 | 4,835 | 10.14 | 1,675 | 9.16 | 5,525 | 9.51 | 985 | 12.75 |
| 2014 | 4,956 | 10.16 | 1,799 | 9.47 | 5,779 | 9.66 | 976 | 12.4 |
| 2015 | 4,815 | 9.94 | 1,930 | 9.36 | 5,646 | 9.26 | 1,099 | 13.72 |
| 2016 | 4,552 | 9.79 | 2,215 | 9.09 | 5,801 | 9.25 | 966 | 11.93 |
| 2017 | 4,802 | 10.44 | 2,444 | 9.29 | 6,163 | 9.64 | 1,083 | 13.13 |
| 2018 | 4,585 | 9.74 | 2,342 | 8.68 | 5,890 | 8.99 | 1,037 | 12.33 |
| 2019 | 4,771 | 9.96 | 2,294 | 8.43 | 6,015 | 9.05 | 1,050 | 12.26 |

SBMs*, synchronous brain metastases.

Rates* were calculated as number of cases per 100,000 person-years and age-adjusted to the 2000 U.S. standard population.

Table S10. SBMs* Incidence-Based mortality Rate* by Sex and Race.

| Year of diagnosis | Sex | | | | Race | | | | | |
|---|---|---|---|---|---|---|---|---|---|---|
| | Male | | Female | | White | | Black | | Other* | |
| | No. cases | Rate | No. cases | Rate | No. cases | Rate | No. cases | Rate | No. cases | Rate |
| 2010 | 1,759 | 6.43 | 1,500 | 4.57 | 2,687 | 5.55 | 371 | 6.41 | 201 | 3.22 |
| 2011 | 2,844 | 10.04 | 2,319 | 6.9 | 4,225 | 8.5 | 597 | 9.96 | 341 | 5.16 |
| 2012 | 3,122 | 10.62 | 2,629 | 7.61 | 4,704 | 9.22 | 659 | 10.44 | 388 | 5.56 |
| 2013 | 3,181 | 10.64 | 2,761 | 7.83 | 4,816 | 9.25 | 664 | 10.29 | 462 | 6.53 |
| 2014 | 3,248 | 10.52 | 2,953 | 8.14 | 5,066 | 9.47 | 651 | 9.83 | 484 | 6.45 |
| 2015 | 3,374 | 10.58 | 2,976 | 8.07 | 5,106 | 9.38 | 707 | 10.23 | 537 | 6.71 |
| 2016 | 3,210 | 9.82 | 2,952 | 7.85 | 4,899 | 8.86 | 716 | 10.19 | 547 | 6.57 |
| 2017 | 3,343 | 10.04 | 3,043 | 7.85 | 5,124 | 9.06 | 743 | 10.09 | 519 | 6.15 |
| 2018 | 3,342 | 9.86 | 2,992 | 7.47 | 5,020 | 8.67 | 714 | 9.55 | 600 | 6.73 |
| 2019 | 3,234 | 9.34 | 2,910 | 7.22 | 4,844 | 8.29 | 713 | 9.37 | 587 | 6.33 |

SBMs*, synchronous brain metastases.

Rates* were calculated as number of cases per 100,000 person-years and age-adjusted to the 2000 U.S. standard population.

Others* included American Indian/AK Native and Asian/Pacific Islander.

Table S11. SBMs* Incidence-Based mortality Rate* according to Age at Diagnosis.

| Year of diagnosis | Age at Diagnosis | | | | | | | |
|---|---|---|---|---|---|---|---|---|
| | 20-39 | | 40-59 | | 60-79 | | ≥80 | |
| | No. cases | Rate | No. cases | Rate | No. cases | Rate | No. cases | Rate |
| 2010 | 27 | 0.18 | 824 | 3.28 | 1,947 | 18.05 | 461 | 16.89 |
| 2011 | 56 | 0.34 | 1,377 | 5.44 | 3,047 | 27.03 | 683 | 24.69 |
| 2012 | 75 | 0.44 | 1,556 | 6.08 | 3,426 | 29.2 | 694 | 24.81 |
| 2013 | 65 | 0.37 | 1,582 | 6.12 | 3,560 | 29.44 | 735 | 26.08 |
| 2014 | 74 | 0.43 | 1,595 | 6.08 | 3,755 | 29.72 | 777 | 27.41 |
| 2015 | 74 | 0.43 | 1,624 | 6.18 | 3,834 | 29.33 | 818 | 28.38 |
| 2016 | 91 | 0.51 | 1,519 | 5.77 | 3,799 | 28.21 | 753 | 25.7 |
| 2017 | 85 | 0.46 | 1,479 | 5.64 | 3,978 | 28.42 | 844 | 28.29 |
| 2018 | 69 | 0.38 | 1,353 | 5.2 | 4,078 | 28.12 | 834 | 27.36 |
| 2019 | 78 | 0.43 | 1,245 | 4.87 | 3,962 | 26.62 | 859 | 27.53 |

SBMs*, synchronous brain metastases.

Rates* were calculated as number of cases per 100,000 person-years and age-adjusted to the 2000 U.S. standard population.

Table S12. SBMs* Incidence Rate* During 2010-2019 According to Median Household Income and Area Distribution.

| Year of diagnosis | Median Household Income | | | | Rural-Urban Distribution | | | |
|---|---|---|---|---|---|---|---|---|
| | <75000 | | ≥75000 | | Urban | | Rural | |
| | No. cases | Rate | No. cases | Rate | No. cases | Rate | No. cases | Rate |
| 2010 | 2,463 | 5.69 | 796 | 4.66 | 2,735 | 5.18 | 524 | 6.99 |
| 2011 | 3,950 | 8.75 | 1,213 | 7.07 | 4,348 | 7.97 | 815 | 10.7 |
| 2012 | 4,498 | 9.42 | 1,253 | 7.64 | 4,814 | 8.56 | 937 | 12.07 |
| 2013 | 4,467 | 9.4 | 1,475 | 8.16 | 5,017 | 8.68 | 925 | 11.97 |
| 2014 | 4,612 | 9.48 | 1,589 | 8.4 | 5,251 | 8.81 | 950 | 12.02 |
| 2015 | 4,584 | 9.46 | 1,766 | 8.57 | 5,336 | 8.76 | 1,014 | 12.65 |
| 2016 | 4,163 | 8.98 | 1,999 | 8.28 | 5,235 | 8.41 | 927 | 11.26 |
| 2017 | 4,349 | 9.45 | 2,037 | 7.74 | 5,375 | 8.4 | 1,011 | 12.24 |
| 2018 | 4,187 | 8.85 | 2,147 | 7.99 | 5,392 | 8.22 | 942 | 11.04 |
| 2019 | 4,172 | 8.7 | 1,972 | 7.21 | 5,146 | 7.74 | 998 | 11.56 |

SBMs*, synchronous brain metastases.

Rates* were calculated as number of cases per 100,000 person-years and age-adjusted to the 2000 U.S. standard population.

Table S13. Incidence and Incidence-Based Mortality of Synchronous Bone, Liver, and Lung metastases without SBMs (2010-2019): the SEER-17registry database.

| Extracranial metastases | Incidence | | Incidence-Based Mortality | |
|---|---|---|---|---|
| | Cases, No. | Rate (95% CI) [a] | Cases, No. | Rate (95% CI) [a] |
| Bone metastases | 150,960 | 12.48(12.42, 12.54) | 115,617 | 9.6(9.55,9.66) |
| Liver metastases | 184,241 | 15.22(15.15, 15.29) | 155,457 | 12.89(12.82,12.95) |
| Lung metastases | 137,857 | 11.44(11.38, 11.5) | 112,596 | 9.38(9.33,9.44) |

SBMs*, synchronous brain metastases.

Rates*, calculated as number of cases or deaths per 100,000 person-years and age-adjusted to the 2000 U.S.

Table S14. Trends in Incidence Rates* of Synchronous Bone, Liver, and Lung metastases without SBMs* (2010-2019): the SEER-17 registry database.

| Extracranial metastases | Overall (2010-2019) | | Trends | | | | | |
|---|---|---|---|---|---|---|---|---|
| | | | 1 | | | 2 | | |
| | APC (95%CI) | P-Value | Year | APC (95%CI) | P-Value | Year | APC (95%CI) | P-Value |
| Bone metastases | 2.0(0.8, 3.3) | < 0.001 | 2010-2017 | 2.9(2, 3.9) | 0.396 | 2017-2019 | -1.1(-7.5, 5.7) | 0.002 |
| Liver metastases | 0.5(0.1, 1) | 0.002 | 2010-2016 | 1.4(0.9, 1.9) | < 0.001 | 2016-2019 | -1.2(-2.6, 0.2) | < 0.001 |
| Lung metastases | 0.2(-0.9, 1.4) | 0.008 | 2010-2016 | 2.3(1, 3.6) | < 0.001 | 2016-2019 | -3.7*(-7.2, -0.1) | < 0.001 |

SBMs*, synchronous brain metastases.

Rates*, calculated as number of cases or deaths per 100,000 person-years and age-adjusted to the 2000 U.S.

Table S15. Trends in Incidence-Based Mortality Rates* of Synchronous Bone, Liver, and Lung metastases without SBMs (2010-2019): the SEER-17 registry database.

| Extracranial metastases | Overall (2010-2019) | | Trends | | | | | |
|---|---|---|---|---|---|---|---|---|
| | | | 1 | | | 2 | | |
| | APC (95%CI) | P-Value | Year | APC (95%CI) | P-Value | Year | APC (95%CI) | P-Value |
| Bone metastases | 15.2(13.6, 16.9) | < 0.001 | 2010-2012 | 57.1(45.3, 69.8) | < 0.001 | 2012-2019 | 5.5(4.4, 6.6) | < 0.001 |
| Liver metastases | 21(18.6, 23.5) | < 0.001 | 2010-2012 | 91.1(71.6, 112.8) | < 0.001 | 2012-2019 | 6.2(4.7, 7.7) | < 0.001 |
| Lung metastases | 21.9(17.3, 26.7) | < 0.001 | 2010-2013 | 42.2(25.4, 61.3) | 0.001 | 2013-2019 | 12.8(8.1, 17.7) | 0.001 |

SBMs*, synchronous brain metastases.

Rates*, calculated as number of cases or deaths per 100,000 person-years and age-adjusted to the 2000 U.S.

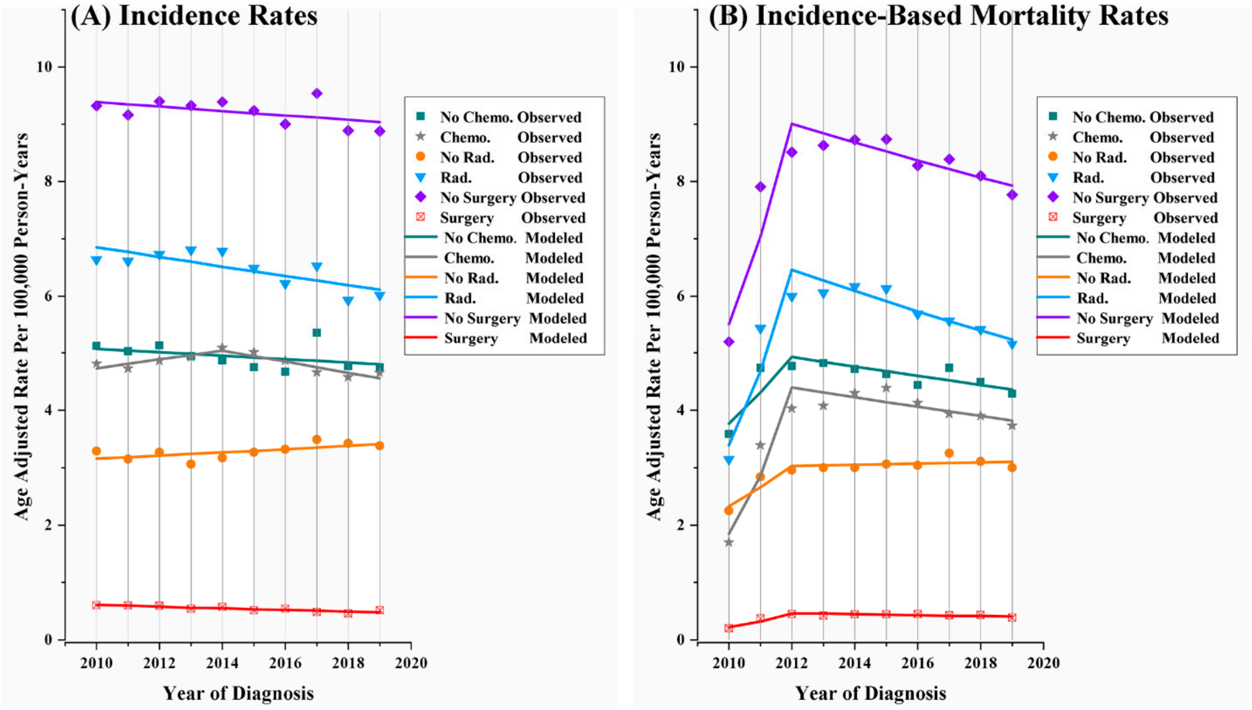


Figure S1. Trends in annual synchronous brain metastases incidence rates (A) and incidence-based mortality (B) by treatment. All rates presented are age-adjusted based on the 2000 U.S. standard population (cases per 100,000 person-years). Each segment on the line represents the annual percent change (APC).

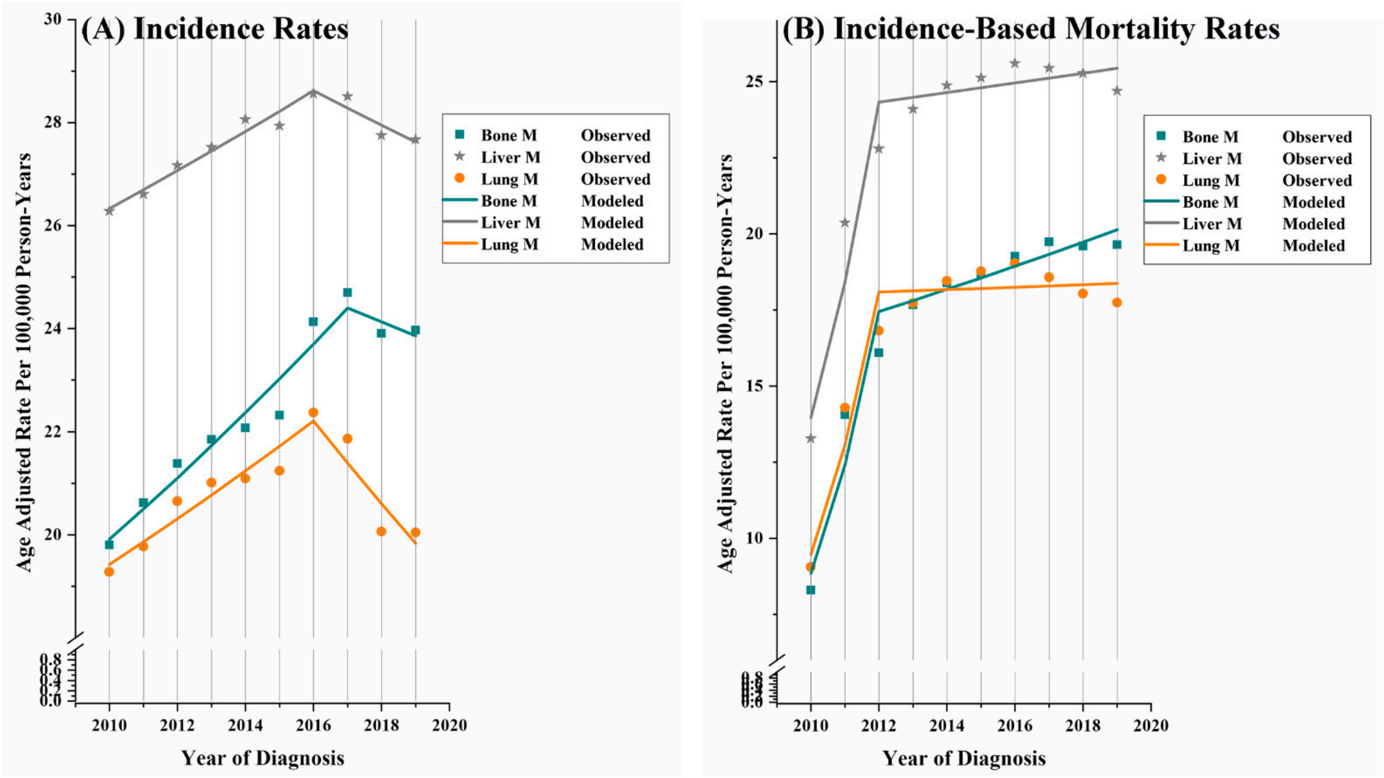


Figure S2. Trends in annual synchronous extracranial metastases incidence rates (A) and incidence-based mortality (B). All rates presented are age-adjusted based on the 2000 U.S. standard population (cases per 100,000 person-years). Each segment on the line represents the annual percent change (APC). Bone M, bone metastases; Liver M, liver metastases; Lung M, lung metastases.
